# Evolving Document Patterns in UK Research Assessment Cycles

**DOI:** 10.3389/frma.2020.00002

**Published:** 2020-04-23

**Authors:** Jonathan Adams, Karen Gurney, Tamar Loach, Martin Szomszor

**Affiliations:** ^1^Policy Institute at King's, King's College London, Virginia Woolf Building, London, United Kingdom; ^2^Institute for Scientific Information, Clarivate Analytics, London, United Kingdom; ^3^Cumberland Infirmary, Carlisle, United Kingdom; ^4^Connected Places Catapult, The Urban Innovation Centre, London, United Kingdom

**Keywords:** research assessment, heuristics & biases, peer - evaluation, peer consensus, Bibliometrics

## Abstract

Researcher behavior is shown to change under assessment. An unexpected time-skew toward most recent papers in each census period was found among the outputs selected by UK academics for the research assessment cycles of the 1990s. This skew changed to a more even time-based distribution for scientists and engineers in later cycles. At the same time, engineers switched their preferred output type for submission, from conference proceedings to journal articles. Social scientists also switched, from monographs to journal art. There was no discussion of these output patterns at the time, or later, but the patterns and their evolution had marked consistency across subjects and institutions. These changes are discussed in terms of consensus and influences on researcher concepts of the evidence of excellence. The increasing availability of citation data in the 1990s and the likely role of citation analysis as a steering factor are noted.

## Introduction

This paper is about the outputs selected by UK researchers for a series of cyclical assessments and the ways in which the pattern of distribution by document type and by year within each census period changed in successive cycles. The data reported are of general interest because the outputs are chosen selectively by the researchers being assessed. The observed skews, and their evolution as more information became available to the researchers, throw light on our understanding of factors influencing expert judgments and status signals among researchers.

This is not a paper about peer review, which has been thoroughly deconstructed elsewhere (de Rijcke et al., 2016), but it has relevance to researcher judgments about research excellence. For peer evaluation to be valid, we assume that a cognate expert group (e.g., researchers in a specific field) share an unwritten set of standards and are competent in using this to judge achievement. If true, then such judgment should apply to choices of material as evidence of research excellence. There are two critical points at which researchers are required to make a judgment about evidence as to whether research is of high quality or significance. One is when they review another researcher's work; the other is when they present evidence of their own achievements. This analysis is about the latter.

The possibility that experienced, informed, expert judgment may in practice contain subjective elements was originally developed in the work of Tversky and Kahneman (1974), who demonstrated the problem in diverse fields including clinical medicine, elite sports, and military recruitment. They proposed that, rather than using only rational decision processes based on objective data, people instead use heuristics—simple experiential rules that focus on a few aspects and exclude others. Three key biases they describe are termed availability (how likely is this?), representativeness (is this object typical of this category?), and adjustment from an anchor (estimates from an arbitrary given point). Heuristics in researcher judgments are noted elsewhere: for example, Bornmann (2015) refers to Gigerenzer and Gaissmeier (2011) who described “recognition bias,” which is a heuristic combining some of the other features. Bornmann (2015) noted the role of heuristics as a source of unconscious, but not necessarily deleterious, bias; Park et al. (2014) noted the potential effect of “herding” in scientific peer review, while Day (2015) also modeled the consequences of small peer review biases in determining funding rates.

In the present study, we look at signals of researcher decision-making in the UK research assessment cycle over the last 25 years. This started with a research selectivity exercise in 1986, became a structured Research Assessment Exercise (RAE) in 1992 (then 1996, 2001, and 2008), and evolved into a Research Excellence Framework (REF) in 2014. Relevant dates are summarized in Table 1 and the detailed history of UK research assessment is reviewed elsewhere (Adams and Gurney, 2010).

**Table 1:** Summary information for the timetable and census period of UK research assessment cycles.

**Cycle**	**Census period**	**Units of assessment**	**Submitted outputs**
1986, Research Selectivity Exercise	Unspecified	37 cost centers	Complete researcher publication lists; no data available
1989	Unspecified	152 subject areas	No data available
1992, Research Assessment Exercise	Arts and humanities January 1988 Other subjects January 1989 Closing date June 30, 1992	72 UOAs	Two publications per researcher plus two other outputs
1996	Arts and humanities January 1990 Other subjects January 1992 Closing date March 31, 1996	69 UOAs	Four selected outputs per researcher
2001	All subjects January 1996–December 2000	69 UOAs	Four selected outputs per researcher
2008	All subjects January 2001–December 2007	67 UOAs	Four selected outputs per researcher
2014, Research Excellence Framework	All subjects January 2008–December 2013	4 panels 36 UOAs	Four selected outputs per researcher

A critical aspect to this analysis is the requirement and ability to freely choose outputs (we are agnostic as to whether it is individuals or their managers that make the final choice, but choice is made). Every assessment requires four outputs per researcher, published during the cycle's census period. Outputs may be books, articles, proceedings, reports, or visual art or performance. The assessment, by peer panels, is based on a comparison against published criteria of excellence (HEFCE, 2014). Since the outcome affects both reputation and funding, it is fair to assume that the material chosen and submitted is deemed to match what peers will recognize as the highest standards of achievement.

Our hypothesis is that a researcher's selection of RAE/REF evidence is based (consciously or not) on a consensus model of academic research excellence. This selection may vary coherently with disciplinary culture but should be consistent across time, institutions, and cognate subjects. It should also be consistent with objective measures of excellence (BIS, 2010; HEFCE, 2014). Our conclusions emerge from the answers to three particular questions:

What material was submitted?
From what sources was that material drawn?
From what times was that material drawn?

The data track changes in the choice of submitted material in later cycles where decisions could be made in the light of information from earlier cycles. There is widespread evidence that the behavior of individuals and organizations responds to assessment, and for this reason, assessment may be introduced to stimulate a particular response. However, behavior may also change in the light of the behavior observed among others (Park et al., 2014), and we suggest that this appears to be the case in this study.

## Data Source

The cycles of UK research assessment are summarized in Table 1 for those unfamiliar with the UK system.

Output data were sourced from the RAE archive sites maintained by the Higher Education Funding Council for England (HEFCE, which managed research assessment for all of the UK higher education funding bodies prior to 2018) and supplemented by prior analytical work on RAE data. The dataset is shaped by the varying length of RAE census periods (the acceptable period for publication cover dates) and by improving technology (e.g., electronic submission).

No data are available for the 1986 and 1989 assessment cycles. The RAE1992 census period ran from January 1988 for arts and humanities, January 1989 for other subjects, and added a part-year with a closing date of June 30, 1992. The RAE1996 census period ran from January 1990 for arts and humanities, January 1992 for other subjects, and added a part-year to March 31, 1996. Data for both are available only at the summary level of subject-based Units of Assessment (UOA).

Census periods for later cycles cover mutually exclusive full calendar years. For RAE2001 (January 1996–December 2000), there is a database of normalized records of submitted outputs. Outputs were not submitted in a standard format but original records could be assigned to output type (book, chapter, article, proceedings, and other). Books and chapters were processed by library staff at the University of Leeds and an ISBN was assigned to each item. Journal articles were processed by *Evidence* Ltd in collaboration with the then Thomson Scientific (now Clarivate Analytics) to clean metadata and assign specific IDs for items indexed in the *Web of Science*™.

RAE2008 introduced a standard electronic format and publication data include Digital Object Identifiers (DOIs). Identifiable, individual records of submitted outputs are thus available for RAE2008 (2001–2007) and REF2014 (2008–2013).

Each assessment cycle received data for some 50,000 UK-based researchers, across 150 higher-education institutions and a gradually reducing number of between 72 and 36 UOAs. The output section of each assessment database (termed the RA2/REF2 section) contains about 200,000 records of outputs submitted as evidence of research achievement at the time. For this analysis, the combined data set of 921,254 outputs selected for assessment is a unique longitudinal perspective on national research activity.

## Methods

Data were initially processed at UOA level (the disciplinary structure of the RAE/REF) and then a set of higher-level categories was used to overcome the problem that UOA count decreased in successive cycles. RAE1992 and RAE1996 data can nominally be reconciled to four REF2014 Main Panels but, where aggregation was required, data were aggregated into domains driven by similarity in publication usage: the analysis underpinning this was originally developed for RAE1996 data and based on clustering UOAs according to similarity in journal frequency (Adams, 1998). This therefore also subsumes broader output differences. The four domains (biomedical and physical sciences; engineering and technological sciences; social and economic sciences; and humanities and visual & performing arts) differ slightly from the REF Main Panel structure, which combines physical sciences with engineering (REF Panel B), leaving biomedical and clinical sciences as a separate entity (REF Panel A).

Data were also analyzed at the level of Higher Education Institutions (HEIs). The data were aggregated to the set of HEIs that existed in 2014 (the date of the most recent REF). The number and structure of HEIs have changed over the period of analysis with new foundations, some mergers (e.g., University of Manchester Institute of Science & Technology merged with the Victoria University of Manchester in 2004), and some splits (e.g., University of London).

Submission selectivity across individual journals was previously tested by us on the RAE2008 data, in unpublished work for the RAE Manager. The frequency with which a journal has articles that have been submitted to the RAE was compared to the available pool for the relevant cycle.

To do this, journal publication records were collated by journal title from the RAE2008 database and compared with the UK pool for 2001–2007. After aggregating all variant titles that occurred five or more times, half of the RAE2008 total journal article submissions (80,829 articles) were collectively accounted for by 669 journals each with 44 or more records. The remaining records occurred in journals at lower frequency including very many singletons of which a high proportion were untraceable.

The relative abundance of articles in the national pool was also analyzed. For example, *Nature* is an international journal with a high profile and a high “impact factor” (Pendlebury and Adams, 2012). The frequency of *Nature* papers with a UK address makes the UK volume in that journal second only to the USA. During the RAE2008 census period of 2001–2007, *Nature* was published every week and contained 18,876 items of which 2,752 (14.6%) had at least one UK address and 1,266 of these were research articles. By comparison, during the same period, the *Journal of Animal Ecology*—a leading serial in zoology and ecology—was published bimonthly and carried 815 items, of which 298 (37%) had a UK address and 286 of these were research articles. So, all other things being equal, while the *Journal of Animal Ecology* is a key serial with a greater UK focus, we expect more records among the outputs submitted to the RAE to be from *Nature*. Note, however, that many UK-authored *Nature* published items were not research articles.

## Results

Results are grouped under six headline outcomes.

### The Pool of Available Material Allows Choice

If researchers are to exercise choice in selecting their RAE/REF submissions, then an over-abundance of source material is required. The data confirm that there is no evidence that a shortage of material constrained the selection of outputs for submission.

First, HEIs reported that submitted outputs for RAE1992 were drawn from a total pool of 787,138 potentially eligible outputs [RAE, 1992–RAE1992–RAE Data RA1–Active Research Staff Return (HEFCE)]. Second, the relatively selective Thomson Reuters *Web of Science* database records 90,000 UK authored journal articles indexed per year so the sum of these across each census period would exceed requirements for that RAE cycle. Third, non-indexed journals, conference proceedings, books, and other forms of output would add to the total pool. The selection of submitted outputs appears therefore to be a deliberate choice, initially skewed toward most recent outputs in RAE1992 and yet subject to modification over successive cycles.

For example, the RAE2008 cycle had a census period from 2001 to 2007. There were about 546,000 papers with at least one UK author among the journals indexed by Thomson Reuters *Web of Science* in that time. Most of these had multiple UK authorships and, even before we add papers for RAE-eligible staff recruited from overseas, that creates many possible submission opportunities. If we add non-journal outputs, then the pool of published material expands to well over three times the required volume to enable selective choices.

### Submitted Output Types Change Over Time

For each RAE cycle, the managing bodies publish a summative report on the research performance of UK institutions and use the indexed outcomes to determine future funding. The reports and funding have a formative influence, encouraging behavior around more, better research (Adams and Gurney, 2010).

**Figure 1:**
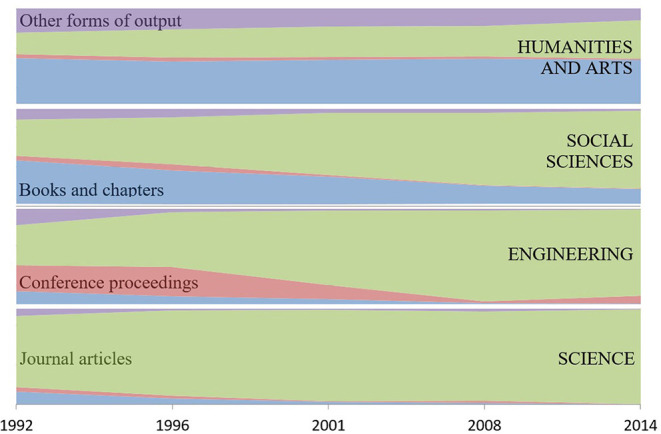
Variation in relative frequency of types of submitted output across successive cycles of the UK Research Assessment Exercise (1992–2008) and Research Excellence Framework (2014). Data are shown for four principal output types and are aggregated for Units of Assessment in four research domains corresponding to the “main panels” of the 2014 exercise. The data show a reduction in diversity with a convergence on journal articles in all areas except Humanities and Arts.

The present data reveal another response: a changing diversity of output types can be observed when the submitted outputs are aggregated at the level of science, engineering, social science, and humanities (Table 2, Figure 1).

**Table 2:** Relative frequency of types of submitted output across successive cycles of the UK Research Assessment Exercise (1992–2008) and Research Excellence Framework (2014).

**RAE1992**	**Science**		**Engineering**		**Social sciences**		**Humanities and arts**	
	**Outputs**	**%**	**Outputs**	**%**	**Outputs**	**%**	**Outputs**	**%**
Books and chapters	5,718	13.2	2,148	13.7	11,463	46	9,553	48.1
Conference proceedings	2,021	4.7	4,207	26.9	1,109	4.5	808	4.1
Journal articles	32,271	74.6	6,650	42.5	9,520	38.2	4,411	22.2
Other	3,258	7.5	2,658	17	2,806	11.3	5,106	25.7
RAE1996								
Books and chapters	5,013	5.8	2,405	8.1	16,185	35.1	22,635	44.4
Conference proceedings	2,657	3.1	9,117	30.8	3,202	6.9	2,133	4.2
Journal articles	77,037	89.8	16,951	57.3	22,575	49	15,135	29.7
Other	1,104	1.3	1,122	3.8	4,154	9	11,128	21.8
RAE2001								
Books and chapters	1,953	2.5	1,438	5.4	12,972	28.6	25,217	46.5
Conference proceedings	751	0.9	3,944	14.9	857	1.9	1,619	3
Journal articles	76,182	95.8	20,657	78.1	29,449	65	17,074	31.5
Other	618	0.8	408	1.5	2,008	4.4	10,345	19.1
RAE2008								
Books and chapters	1,048	1.2	216	1.2	12,632	19	21,579	47.6
Conference proceedings	2,164	2.5	326	1.8	614	0.9	897	2
Journal articles	80,203	93.8	17,451	95.4	50,163	75.5	14,543	32.1
Other	2,125	2.5	301	1.6	3,018	4.5	8,287	18.3
REF2014								
Books and chapters	228	0.3	197	0.8	8,307	15.9	18,168	46.3
Conference proceedings	81	0.1	2,056	7.9	233	0.4	380	1
Journal articles	73,039	99.1	23,521	90.9	42,545	81.5	15,749	40.2
Other	331	0.4	108	0.4	1,105	2.1	4,914	12.5
REF case studies								
Books and chapters	274	2.1	282	6.3	1,819	16.9	3,409	40.0
Conference proceedings	150	1.2	686	15.4	195	1.8	334	3.9
Journal articles	11,752	91.7	3,263	73.4	7,102	66.0	3,251	38.1
Other	631	4.9	213	4.8	1,649	15.3	1,523	17.9

*Data cover four principal output types and are aggregated for Units of Assessment in four research domains corresponding to the “main panels” of the 2014 exercise. The data are visualized in Figure 1*.

The format for RAE1992, which established the system of four outputs per person, was published only in March 1992 so the first cycle's data should not be over-interpreted. RAE1992 required only two publications per researcher (plus two other outputs) so there are relatively more books in science and engineering, though the absolute number is similar to RAE1996. Nonetheless, for 1992 and 1996, it is apparent that a preference (in the sense of making a relatively frequent selection) existed for journal articles among scientists; engineers preferred proceedings; the social scientists and scholars in the humanities preferred the monograph; while visual and performing arts used specialized media.

Neither the RAE Manager nor any UOA panel ever published advice indicating a preference for particular types of output. Nonetheless, evidence of change emerged as early as RAE1996, with engineers' preference shifting from conference proceedings and social scientists shifting from books (i.e., publication modes common for those disciplines in RAE1992) toward journal articles. The shift went further in 2008 and in 2014 so that journal articles became the predominant submitted output in all areas except humanities, which remain focused on books. The humanities/arts shift in frequency from “other outputs” to “journal articles” between 2008 and 2014 is driven by changes in arts researchers' submitted outputs.

### High-Impact Journals Are Preferred to Highly Cited Articles

There were 14 journals (“frequently submitted journals”) that each had more than 500 RAE2008 output records, and these accounted for 8.5% of journal outputs. Three of the most frequent journals (*Nature, The Lancet*, and *Science*) were present with a greater number of records than the number of unique UK-addressed papers (i.e., articles and reviews) published in the census period. One *Nature* paper was present in the RAE2008 database as 12 separate records: the authors were from 12 different UK universities and each institution submitted that same paper once.

A common factor for the journals with an exceptional RAE/UK submission ratio (where records submitted exceeded papers published) is their average citation impact: in each case, the Thomson Reuters “journal impact factor” exceeded 30. For other journals with over 500 RAE2008 output records, with the exception of the *British Medical Journal* at 14, the journal impact factor was high but did not exceed 10.

**Figure 2:**
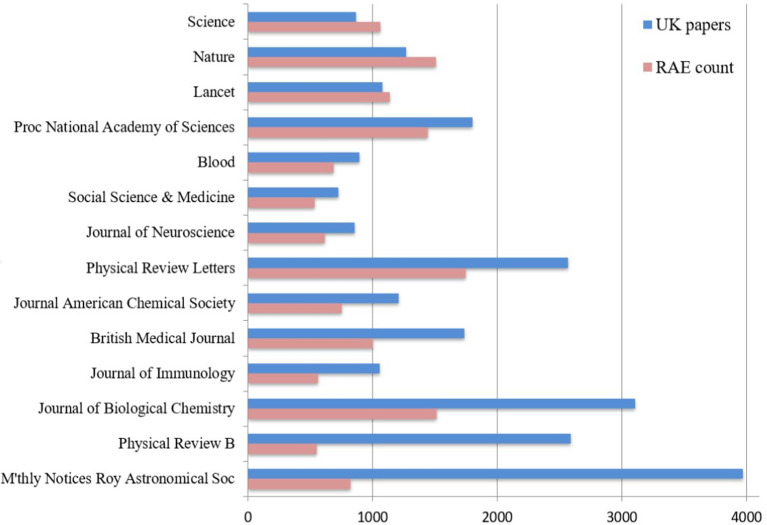
Frequency of articles in a range of journals with a high citation impact factor. “UK papers” is the number of articles with at least one UK author-address during the period 2001–2007 (the census period of RAE2008). “RAE count” is the number of articles submitted to the Research Assessment Exercise in 2008.

There were 68 other journals submitted at a high ratio (>1.0) compared to the availability of UK-authored papers, and 29 had more than 100 RAE2008 records. In addition to the 3 with over 500 records (Figure 2), the other 26 fell into two distinct groups: 13 science and medicine journals, of which 9 had journal impact factors >10; and 9 business, management and economics journals that also had high journal impact factors relative to their field. The four other journals with a high submission ratio cross diverse subjects: The *Historical Journal* (133 RAE records); *Theoretical Computer Science* (154); *Regional Studies* (184); and *Human Relations* (188).

For journals frequently submitted to the RAE, it is unlikely that all submitted items exceed the average citation impact because of the typically skewed distribution of citations to articles. Typically, about two-thirds of the papers will have less than the average citation count because of a smaller proportion of exceptionally highly cited items.

*Nature* had 1,510 RAE2008 records and 1,266 UK-addressed articles published in the census period, so it was possible that every eligible UK paper was submitted. In fact, only 740 unique RAE papers could be matched to a *Nature* article or review, so 500 eligible UK *Nature* papers were not submitted. On the other hand, some of the 740 papers that were submitted appeared several times, so they cover 1,092 submission records. The remaining 418 RAE2008 records drawn from *Nature* cannot be matched to UK articles and reviews either because (in a few cases) they were authored by researchers recruited from outside the UK or because (for the majority) they were some of the 1,486 letters, editorials, corrections, and other pieces published by *Nature* between 2001 and 2007 that had UK addresses.

Among the 740 matched *Nature* papers, more than a dozen are cited over 1,000 times and clearly mark outstanding pieces of research. However, over 100 papers were cited by less than one-quarter of the average number of citations for their *Nature* volume. When a sample of such papers was checked at an individual author level, it was evident that some researchers had eligible papers from other journals that had not only more citations but also a better relative impact for their discipline.

### Skewed Publication Dates for Submitted Outputs Changes in Later Cycles

The publication dates of RAE-submitted outputs are skewed toward the more recent years in each cycle. The time-skew appears to be a general phenomenon.

All other things being equal, the spread of submitted outputs by time within a RAE census period might be uniform. Contrary to this, RAE1992 data reveal a very marked time-skew of submitted outputs toward the most recent publication dates for that cycle (1992 was a “half-year” with a census cutoff at 30 June). This skew persisted in RAE1996 and later cycles, but this analysis shows that it gradually moderated. Note that a change in the number of years in each cycle (from a core of 4 years to 5 to 7 to 6) affects the height of the curve and that two—not four—publications plus “two other outputs” were required per researcher in RAE1992.

**Figure 3:**
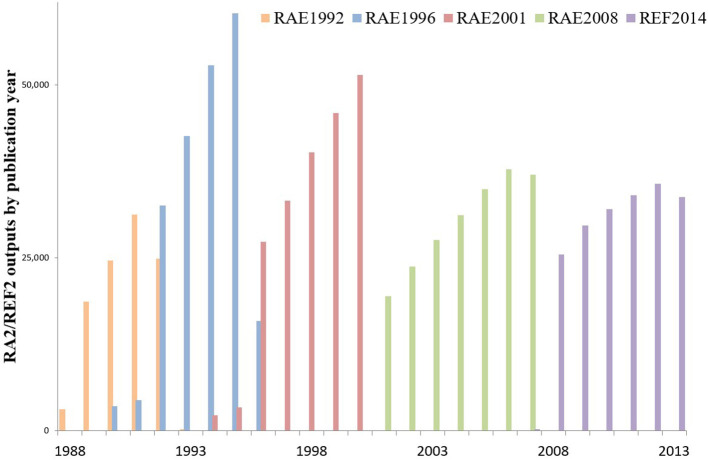
The total number of outputs submitted to successive cycles of the UK Research Assessment Exercise (1992–2008) and Research Excellence Framework (2014). Data are aggregated by year of publication. The RAE1992 census included the part-year of 1992 to the June submission deadline. In RAE1996, submissions were included to 31 March 1996. Other cycles used only whole calendar years. Humanities submissions were allowed for two additional years, back to 1990 in RAE1996 and back to 1994 in RAE2001.

The last full year of the census period provided the greatest number of publications submitted for assessment in RAEs 1992, 1996, and 2001. RAE1992 allowed publisher-accepted outputs in the year of assessment and RAE1996 allowed submissions to March 1996, but 1991 and 1995 are the peak publication years: 1995 has twice the output volume of 1992, the first year of the RAE1996 cycle, and, in RAE2001, 2000 has almost twice the volume of 1996. In RAE2008, however, the penultimate year (2006) is the peak publication point for submitted outputs. In REF2014, the time profile is more evenly distributed and 2011 and 2012 publication volumes both exceeded 2013 (Figure 3).

The pattern was not reported by RAE administrators, nor was it recognized by university research managers. Several senior research and institutional managers were consulted about the results reported in this paper: they confirmed that the recency skew in submission publication dates was unknown to them, had not been reported in institutional data, and was not discussed in subject-based conferences. It is relevant to note that a common external factor at this time will have been increasing awareness of citation data.

### Behavior in Selecting Submitted Outputs Is Consistent Across UOAs and HEIs

The net values for the whole database in each cycle (Figure 3) capture the aggregate of many independent HEI and UOA submission choices. The expressed preferences may be homogeneous and generic or the profile may be the smoothed outcome of combining many heterogeneous variants from disciplines with varying cultures and from HEIs with different missions.

To explore whether the observed profile is homogeneous across UOAs and HEIs, two comparative analyses were created by calculating the percentage of total outputs by publication year for each UOA in each cycle and for each of the 150+ submitting HEIs. The median value in each year and the upper and lower quartiles bounding that median indicate the spread across units (UOAs and HEIs).

**Figure 4:**
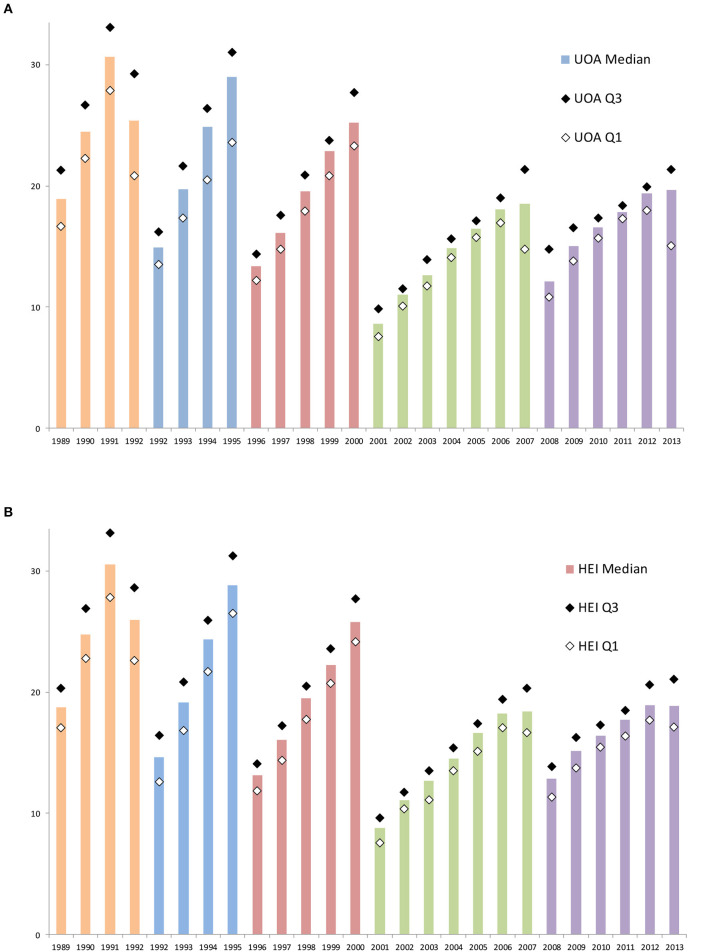
The share of outputs submitted to each year of a census period in successive cycles of the UK Research Assessment Exercise (1992–2008) and Research Excellence Framework (2014). Data are aggregated by year of publication and shown as a percentage of the total submissions for that cycle. **(A)** The median and the lower (Q1) quartile and upper (Q3) shares for data aggregated at the level of subject-based Units of Assessment (UOA). **(B)** The median and the lower (Q1) quartile and upper (Q3) shares for data aggregated at the level of submitting Higher Education Institutions (universities and colleges, HEIs).

This analysis reveals that the 1989–2013 profiles for UOAs (Figure 4A) and HEIs (Figure 4B) are similar both to one another and to the overall picture (Figure 3). There is remarkably low variance, with quartiles around the median that are relatively tightly bounded compared to the year-to-year change in medians, and with a narrowing interquartile spread in later cycles.

Recall that these submitted outputs are the combined selections of four outputs for each of 50,000 individual researchers across institutions and subject areas. As noted, the skew was not reported at the time and managers recalled no awareness. Nonetheless, many independent choices lead to a well-defined time-skew, consistent across disaggregated UOA and HEI units, that then moderates in a coherent way for those subjects and institutions across cycles.

### Science/Technology and Social Science/Arts Make Different Choices

Initial cultural distinctions in the selection of outputs submitted for assessment are displaced by a convergence on journal articles (Figure 1). However, the analyses underpinning Figure 3 provide statistical information that throws light on cultural divergence in other aspects of behavior.

For outputs submitted by year of publication, the average across the dataset is lower than median values for individual UOAs and HEIs. This is due to a volume factor, where the relatively large volumes of submitted outputs for a small number of UOAs influence the overall average. The disparity implies UOA-related differences that affect HEIs according to the subject balance of their portfolio. There is also greater variance toward the end of later cycles (the interquartile range of medians increases), implying an emerging difference.

**Figure 5:**
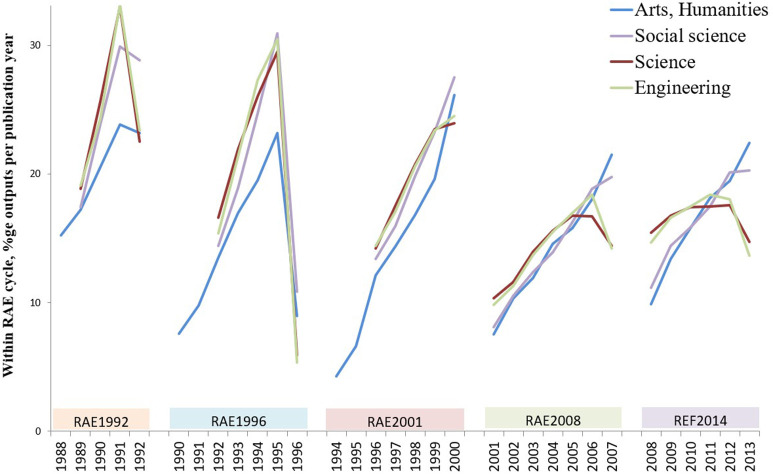
The share of outputs submitted to each year of a census period in successive cycles of the UK Research Assessment Exercise (1992–2008) and Research Excellence Framework (2014). Data are aggregated by “main panels” as principal research domains and shown as a percentage of the total submissions for that cycle.

To explore the source of these disparities, the data were re-aggregated into the four broad subject groups (medical and natural sciences; engineering and technology; social sciences and business; and humanities and arts, as in Figure 1). At this group level (see Figure 5):

In RAE1992, profiles are similar but humanities and social sciences are more “recent” to a similar extent, with the 6-month 1992 output count approaching the full year of 1991.
In RAE1996, the profiles appear similar across subject groups (the humanities line is lower only because of data spread across two additional census years).
In RAE2001, a slight terminal inflection appears for science and engineering.
In RAE2008, a divergence between “arts” and “sciences” becomes clear.
In REF2014, science and engineering shift profile to peak across two penultimate years and dip in the final census year while social sciences and humanities/arts retain the skew of earlier cycles.

“Sciences” chose less recent submitted outputs than “arts” in the early cycles and then later shifted further toward a more even spread across years, a change that does not occur in social sciences or in humanities. It should be recalled that this is the outcome of many independent submission choices and yet these choices were coherently and systematically modified. This pattern, with a separation between “science/technology” and “social science/humanities” is unexpected, as is the marked similarity within each pair but it is consistent with the idea that growing awareness of citation data may have been influential.

## Discussion

Researchers are expected to make informed choices about representative evidence of excellence in their outputs, based on cultural standards shared with peers. The analyses in this study were based on researcher selection of submitted RAE and REF outputs.

The data show that there were always more than enough UK-authored outputs available in each assessment cycle. The majority of researchers will have been able to pick and choose their “best” outputs, although some individual choices may have been constrained. However, the pattern of choices changed over a period when more information about citations, bibliometrics, and quantitative research assessment was becoming available. It seems obvious to conclude that research-based judgments have become subject to citation-based data.

The analytical evidence addressing the three questions posed in the *Introduction* is, in summary, that there is a peer consensus that is in practice guided by heuristics (experiential rules) and that basis of choice is not a fixed cultural reference but is subject to modification in the light of decision-making observed among others (herding *sensu* Park et al., 2014) including the data published after earlier cycles.

- What material was submitted?

The most frequent submitted outputs were consistently journal articles for natural scientists. Initially, engineers and social scientists submitted, respectively, conference proceedings and monographs but they modified their choices in successive cycles and switched increasingly to journal articles. Evidence initially preferred on a diverse cultural basis was thus displaced by a convergent focus on journals as a privileged output type (Figure 1).

- From what sources was that material drawn?

The sources of articles were disproportionately often journals with a relatively high average citation impact. Such selections were made even where individuals had more highly cited outputs in lower-impact journals (Figure 2). Evidence driven by a generic source indicator was thus preferred to evidence based on specific achievement.

- From what times was that material drawn?

Submitted outputs were initially drawn from (or “skewed” toward) the most recent years in each census period. This skew was very consistent across subject areas and institutions. The skew diminished in successive cycles for natural sciences and engineering and at the same pace, but no such change occurred for social sciences and humanities/arts. Evidence preferred on an assumption of recent achievement was thus replaced (appropriately?) by evidence of earlier and demonstrable achievement (Figure 3).

If these rules hold for researchers' own choices of material as evidence of excellence about themselves, then we may infer that this is also a general property of their assessment of evidence about others. In other words, while academics believe their judgment rests on evidence-based experience, their peer review may also be influenced by heuristics and biases as has been shown for other expert groups (Tversky and Kahneman, 1974).

The identification of these heuristics does not detract from the confirmation of a marked and pervasive peer consensus. The acceptability of peer review depends on such a consensus. In this regard, the analysis of publication dates shows a remarkable conformity with a distinctive time-skew. The skew is toward the most recent publication year in each cycle. The national variation in the share by year is so low that the interquartile spread of shares barely overlaps between successive years. This suggests that, despite any discussion, the submissions of 50,000 researchers across 72 UOAs (Figure 4A) and 150 HEIs (Figure 4B) tended to be drawn at rather similar rates from different years in a cycle. This implies a powerful, common conceptual model regarding optimal material.

Why the (initial) skew to recency? The simplest explanation would be that researchers believe they are only as good as their last publication. Despite this initial consensus, there is a change and departure from the initial skew over time across successive cycles. The change occurs only among the scientists and the engineers, but when they change, they do so at a similar rate. The initial consensus is not a fixed template; a change in skew is not universally compelling, but where change occurs, it is common to diverse disciplines. What drives these evolving choices? The most likely explanation is the availability of citation data.

There are two patterns of change. One is the shift from recent publications among scientists and engineers toward a more even time spread. The second is the shift among engineers from conference proceedings and among social scientists from monographs, in both cases toward journal articles. These may both be driven by the increasing availability and deployment of citation data through the 1990s. Citation analysis is applied specifically to journal articles, particularly through the *Web of Science*, enabling analysts to develop indices of more or less impactful work. In 1990, citation analysis was hardly used in public research policy. In the mid-1990s, it was drawn to wider attention (May, 1997; Adams, 1998). By 2000, it had become a widely discussed tool at national and institutional level (Adams and Smith, 2002). Two things follow: first, assigning an index to the “academic impact” of an article may imply a primacy over items (books, proceedings) not so quantified; second, researchers may review their own publications to discover which have delivered greater “impact.”

The change in the diversity of submitted material, toward journal articles in engineering and social sciences, is driven by the “quantitative prioritization” that assigns a citation value to one type of output and not to others. That value obscures, or overrides, a prior cultural sense of what represents the more significant research publication. The change in skew, toward a more even spread across the census period, is driven by feedback on observed article citations. A researcher now sees that older publications have received attention and acknowledgment, conferring a tangible index of excellence ahead of their latest idea.

The evolving choices, away from recency and toward journals, are evidence that researchers are as susceptible to heuristics as other expert groups, and also adaptable in their judgments as their assumptions meet real data. There are no “wrong” choices since the view at any time continues to be a consensus one, but the decisions about assessment grades made in the earlier cycles will not necessarily be entirely consonant with those made more recently.

## Data Availability Statement

The researcher output submission data analyzed in this study was obtained under a series of contracts from the Higher Education Funding Council for England on behalf of the RAE Manager. Requests to access these datasets should be directed to UK Research & Innovation, Polaris House, Swindon SN2 1FL. Additional metadata for article records were sourced from the Web of Science, which is accessible to academic researchers in the UK under licence from the Joint Information Services Committee and in other countries through separate licensing agreements.

## Author Contributions

The authors contributed equally, though to different phases of the work and over several assessment cycles.

## Conflict of Interest

JA and MS are employed by Clarivate Analytics, the parent company of Web of Science and the Institute for Scientific Information. The remaining authors declare that the research was conducted in the absence of any commercial or financial relationships that could be construed as a potential conflict of interest.
